# Genetic risk stratification and risk factors of early menopause in women: a multi-center study utilizing polygenic risk scores

**DOI:** 10.3389/fendo.2024.1518288

**Published:** 2024-12-02

**Authors:** Wei Zhong, Qihang Wang, Dingchuan Peng, Yangyun Zou, Yulin Chen, Yingying Xia, Xin Zhang, Mingming Shu, Chunlan Song, Yiran Wang, Yiyao Fu, Sishuo Wang, Yanmin Ma, Xiaomeng Bu, Yuexiu Liang, Yuzhen Chen, Wenpei Bai, Yanrong Chen, Chengyan Deng, Wanyu Zhang, Ming Zhou, Lijuan Lv, Linyan Zhang, Sijia Lu, Wei Shang

**Affiliations:** ^1^ Department of Obstetrics and Gynecology, The Seventh Medical Center of People’s Liberation Army General Hospital, Beijing, China; ^2^ Department of Obstetrics and Gynecology, The Sixth Medical Center of People's Liberation Army General Hospital, Beijing, China; ^3^ Department of Obstetrics and Gynecology, Beijing Obstetrics and Gynecology Hospital, Capital Medical University, Beijing, China; ^4^ Department of Clinical Research, Yikon Genomics Co., Ltd, Suzhou, Jiangsu, China; ^5^ Department of Obstetrics and Gynecology, Chinese People's Liberation Army Medical School, Beijing, China; ^6^ Department of Obstetrics and Gynecology, Affiliated Hospital of Youjiang Medical University of Nationalities, Baise, Guangxi, China; ^7^ Department of Obstetrics and Gynecology, BeiJing Shijitan Hospital, Beijing, China; ^8^ Department of Obstetrics and Gynecology, Peking Union Medical College Hospital, Beijing, China; ^9^ Department of Obstetrics and Gynecology, Huanghua Development Zone Boai Hospital, Cangzhou, Hebei, China; ^10^ Department of Obstetrics and Gynecology, Maternal and Child Health Hospital of Zhuji, Zhuji, Zhejiang, China

**Keywords:** early menopause, premature ovarian failure, polygenic risk scores, premature ovarian insufficiency, multi-center

## Abstract

**Objective:**

This study aims to evaluate the utility of polygenic risk scores (PRS) in women with early menopause (EM) and to investigate the clinical characteristics and risk factors associated with EM based on genetic risk.

**Study design:**

Genotyping data and clinical data from women with EM and women with normal age of menopause retrieved from UK Biobank were used for early menopause risk prediction model establishment. Subsequently, 99 women diagnosed with EM and 1027 control women underwent PGT-M were recruited for model validation from across eight hospitals in China. According to PRS percentiles, these participants were further classified into high risk and intermediate risk groups. Characteristics among women at different risk levels were compared, and risk factors with early menopause were also statistical analyzed.

**Main outcome measures:**

The proportion of women at high risk in EM and control groups; Characteristics with significant difference among women at different risk levels; risk factors associated with EM.

**Results:**

The proportion of high-risk women in the EM group was significantly higher than that in control women underwent PGT-M (Group PGT-M) (OR = 3.78), and that in women with normal age menopause from UK Biobank (Group UKB) (OR = 5.11). Notably, the women with high risk of EM exhibited distinct characteristics compared to women with the intermediate-risk of EM, and identified several risk factors associated with EM.

**Conclusions:**

We established a PRS model to serves as a valuable instrument for EM risk prediction. The exploratory analysis revealed that women with high risk of EM exhibited a higher height, suggesting EM related genetic loci may also influence growth and development level. Several risk factors were found to be potentially associated with EM, such as excessive familial contentment, COVID-19 vaccination, staying up late, and the husband’s engagement in smoking and alcohol abuse.

## 1 Introduction

In contrast to the delineation of premature ovarian insufficiency (POI), early menopause occurs in women prior to their forty-fifth year (1). The etiology of early menopause is complex and multifaceted, resulting in adverse effects on women’s health (2) and professional endeavors (1). Consequently, the ability to forecast the advent of early menopause in younger women is of paramount societal importance, particularly in the context of preserving fertility. Within the purview of the present investigation, the variables utilized to anticipate the manifestation of unexplained early menopause predominantly encompass exogenous elements (including environmental influences, lifestyle choices, and social determinants) and serological indices.

Research suggests that both genetic and exogenous factors play a role in the onset of early menopause. Genetic influences, such as a family history of early menopause, specific genetic variants, and the heritability of menopausal age, have been identified as significant predictors (3, 4). Social environmental factors, including early menarche, nulliparity, low parity, cigarette smoking, and being underweight, have also been linked to an increased risk of early menopause (3). These discoveries underscore the intricate interconnection between genetic factors and social environment in precipitating early menopause.

Nevertheless, numerous studies attempting to predict the onset of early menopause have concentrated mainly on exogenous factors, with a notable absence of consideration for genetic factors. This oversight not only results in inaccurate predictions but also impedes the investigation of environmental factors that influence the onset of early menopause due to the confounding influence of genetic factors. For example, a certain proportion of non-smokers may experience early menopause due to genetic predisposition. This results in a reduction in the statistical efficacy of comparing the risk factor of smoking with that of women experiencing regular menopause.

Polygenic risk scores (PRS) represent a method for predicting an individual’s genetic susceptibility to diseases. They are calculated by evaluating the presence of risk variants identified in genome-wide association studies (5). While PRS, in isolation, cannot definitively predict a diagnosis, they can be integrated with additional risk factors to enhance the prediction of outcomes and support clinical decision-making. The PRS has been employed in several studies, including an investigation into its potential association with early menopause (6). The utilization of PRS models for early menopause enables the classification of women into groups based on genetic susceptibility, facilitating more targeted research on factors influencing menopausal events. Nevertheless, there remains a scarcity of studies and limited investigation into the predictive capabilities of PRS models for early menopause within East Asian populations.

In this investigation, women experiencing EM were recruited from a multicenter study in China and classified into two distinct groups based on their genetic susceptibility, as determined by the PRS model. This methodology was adopted to investigate the disparities in attributes between these cohorts and to scrutinize the risk factors implicated in EM. The overarching aim is to determine the efficacy of the PRS model as a prognostic instrument for EM and to unveil risk factors inherent in exogenous factors for early menopause.

## 2 Materials and methods

### 2.1 Participants

**Figure 1:**
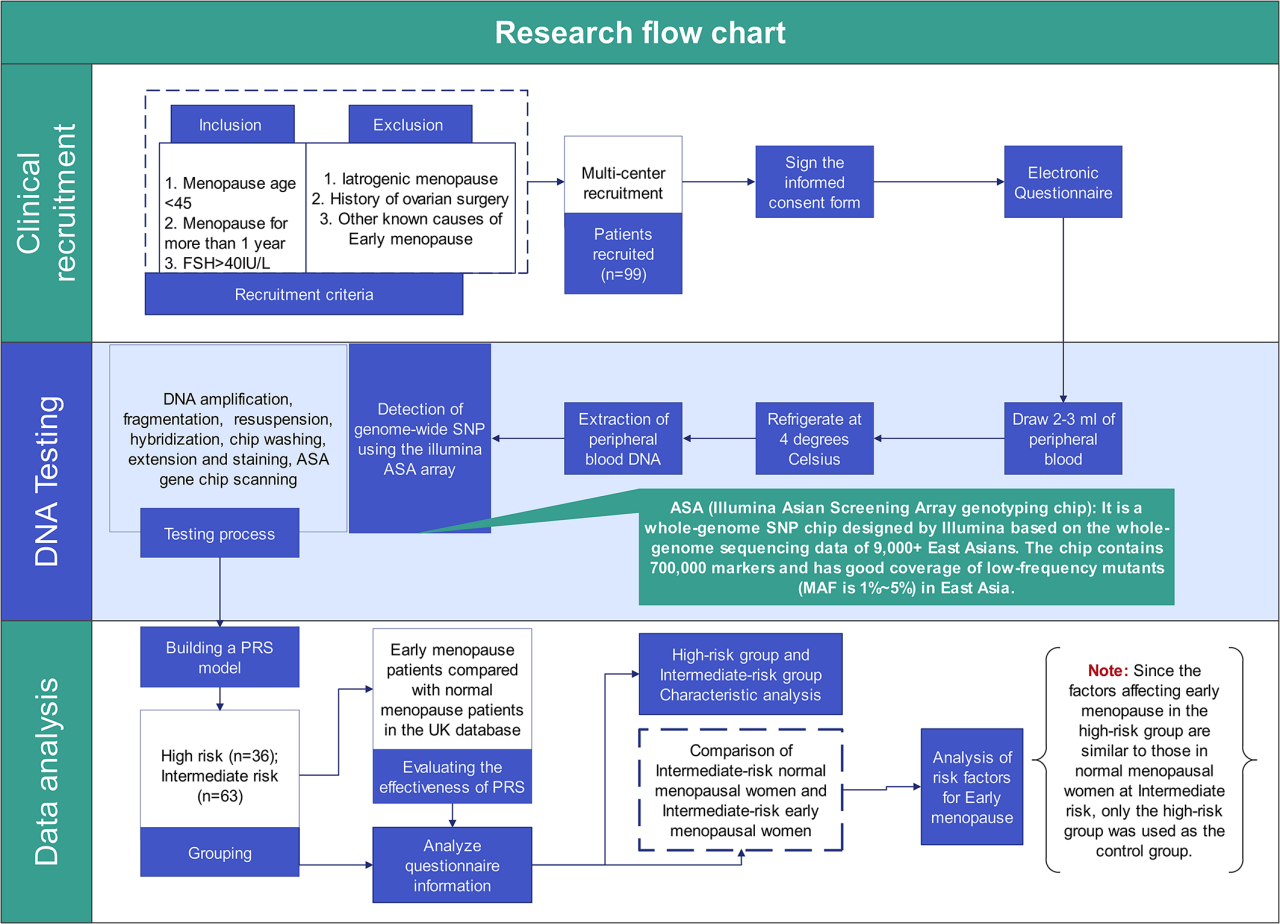
The research design flow chart. This Figure shows the complete process and work details of this study from patient recruitment to data analysis.

Two sets of participants were included in the present study. For EM risk prediction model establishment, 154,320 women with known age of menopause derived from UK Biobank (https://www.ukbiobank.ac.uk/). Among them, 20,408 women were found with age of menopause earlier than 45 years old, namely EM. For EM risk model validation, 99 Chinese women with EM and 1027 Chinese control women, who previously underwent PGT-M, were recruited from eight hospitals in China. Besides, 368 Chinese women with normal age of menopause from UK Biobank were further retrieved and used as healthy controls. **Figure 1** shows the overall design of the study.

### 2.2 Patients with known age of menopause from UK Biobank

The UK Biobank is a large prospective cohort study that enrolled individuals from across the United Kingdom, aged 40-69 years at the time of recruitment, starting in 2006. In this cohort, 154,320 women were found with known age of menopause. Among them, 20,408 women were with age of menopause earlier than 45 years old (EM). For EM PRS model establishment, genotyping microarray genetic data and clinical information from these individuals were retrieved. Besides, 368 Chinese women with normal age of menopause from UK Biobank were further retrieved and used as healthy controls.

### 2.3 Recruitment of Chinese patients with EM

This study commenced recruiting patients with unexplained early menopause from 2022 to 2024, conducted across eight hospitals in China. The research complies with the Declaration of Helsinki and was approved by the Ethics Committee of Chinese People’s Liberation Army, General Hospital (2023-XJSXYW-003). All participants signed informed consent forms, acknowledging the study’s significance in understanding genetic risk factors for early menopause. For married women, spousal consent was also obtained due to the need for personal information.

The inclusion criteria encompass women with menopause at or before the age of 45, patients diagnosed with premature ovarian failure (POF) confirmed by two follicle-stimulating hormone (FSH) measurements exceeding 40 IU/L, and women undergoing long-term hormone replacement therapy to maintain menstruation. Patients diagnosed with primary ovarian insufficiency (POI) whose FSH levels do not reach 40 IU/L are excluded since it is indeterminate whether they experienced menopause before age 45. Recruitment is conducted through outpatient clinics in multiple hospitals and online platforms. Participants also completed an electronic questionnaire covering personal habits, environmental factors, and medical history related to early menopause. The questionnaire was designed with mandatory fields to minimize missing data, except for non-applicable items (e.g., spousal information for unmarried women). Such non-applicable data were excluded from the analysis.

Participants are excluded from the study if they meet any of the following criteria: 1. They have not reached early menopause or POF status. 2. They have identifiable causes for early menopause, such as radiotherapy, chemotherapy, oophorectomy, or genetic conditions (monogenic or chromosomal abnormalities). 3. They refuse to participate in the questionnaire survey.

### 2.4 Recruitment of Chinese women previously underwent PGT-M as controls

Due to lack of Chinese women with normal age of menopause as healthy controls, we compromised to use Chinese women previously underwent PGT-M as general population controls. The age of menopause of these women were unknown because of not yet of timing or unobtainable due to lack of information. Thus, we considered these women as general population with a proportion of women potentially with EM in the future. Finally, 1027 women previously underwent PGT-M were recruited from various hospitals. The genotyping microarray genetic data using Illumina’s Infinium Asian Screening Array (ASA) of each woman was retrieved and analyzed.

### 2.5 SNP genotyping

Ethylenediaminetetraacetic acid (EDTA) Peripheral blood (5 mL) was collected from 99 Chinese women with EM. Genotyping of these women was performed with the Illumina’s ASA genotyping chip. Quality control of DNA sample, genotyping call rate, and genotype imputation were performed according to previously described methods. Samples found to be genotypically not female, discordant, or cryptic duplicate pairs were excluded. SNPs with assay call rate<90%, deviation from Hardy–Weinberg equilibrium (p-value<10^−7^ for controls or p-value<10^−12^ for cases) were excluded. Data were imputed using BEAGLE with 1000 Genomes Project (Phase 3) as reference panel. Only non-monomorphic SNPs in East Asian population in the reference panel were imputed. SNPs with an imputation accuracy score<0.9 were excluded.

### 2.6 Establishment of EM PRS model based on UK biobank dataset

An EM PRS model was developed using 290 SNPs and corresponding weight derived from previous GWAS results (7). The algorithm applied for PRS calculating was as following:


$$PRS=\beta_{1}\times SNP_{1}+\beta_{2}\times SNP_{2}+\cdots +\beta_{\text{n}}\times SNP_{\text{n}}$$

where *SNP_n_* is the SNP locus and is assigned with value 0, 1, and 2, corresponding to the homozygous non-risk allele, the heterozygous risk allele and the homozygous risk allele respectively; *β_n_* is the weight of *SNP_n_* associated with EM associated. Based on the UK Biobank genotyping microarray genetic data for 154,320 women with known age of menopause, including 20,408 women with EM, we calculated EM PRS for each individual and constructed a baseline distribution of PRS in the European population. Combining age of menopause information of each woman, we evaluated the relationship between the PRS percentile and the proportion of women with EM within each PRS percentile.

### 2.7 Validation of EM PRS model in Chinese cohort

A Chinese cohort with 99 women with EM and 1027 women as general population controls were used for EM PRS model validation. PRS was calculated for each woman and the proportion of women with EM within each PRS percentile was further analyzed. The performance of the EM PRS model was evaluated by the area under the receiver operator characteristic curve (AUC), and the cut-off of PRS percentile to define a woman with predicted high risk of EM was obtained, accordingly.

### 2.8 Statistical methods

For the analysis of differences between two groups of quantitative data, we used the Student’s t-test for two independent samples. For the comparison of count data, we employed the Pearson chi-square test. When the expected frequency in any cell is less than 5, the results of the chi-squared test may be unreliable, in which case Fisher’s exact test should be used. A p-value of less than 0.05 was considered statistically significant for all tests.

All statistical analyses were conducted using R software (version 4.2). The primary objective of this study was to validate the applicability of a PRS model in Chinese female populations. The odds ratios were employed as the measure of effect size to evaluate the discriminatory capacity of the PRS models when applied respectively in the EM population and the control groups.

Moreover, the main purpose of the study was not designed to investigate risk factors associated with EM, and as such, a strictly defined control group with corresponding clinical information was not established. Additionally, potential confounding factors were not controlled for in the statistical analysis, as controlling for these variables was beyond the scope of the current study design. Genetic risk comparisons across different groups were performed as exploratory analyses.

### 2.9 Polygenic risk score stratification in menopausal group

**Figure 2:**
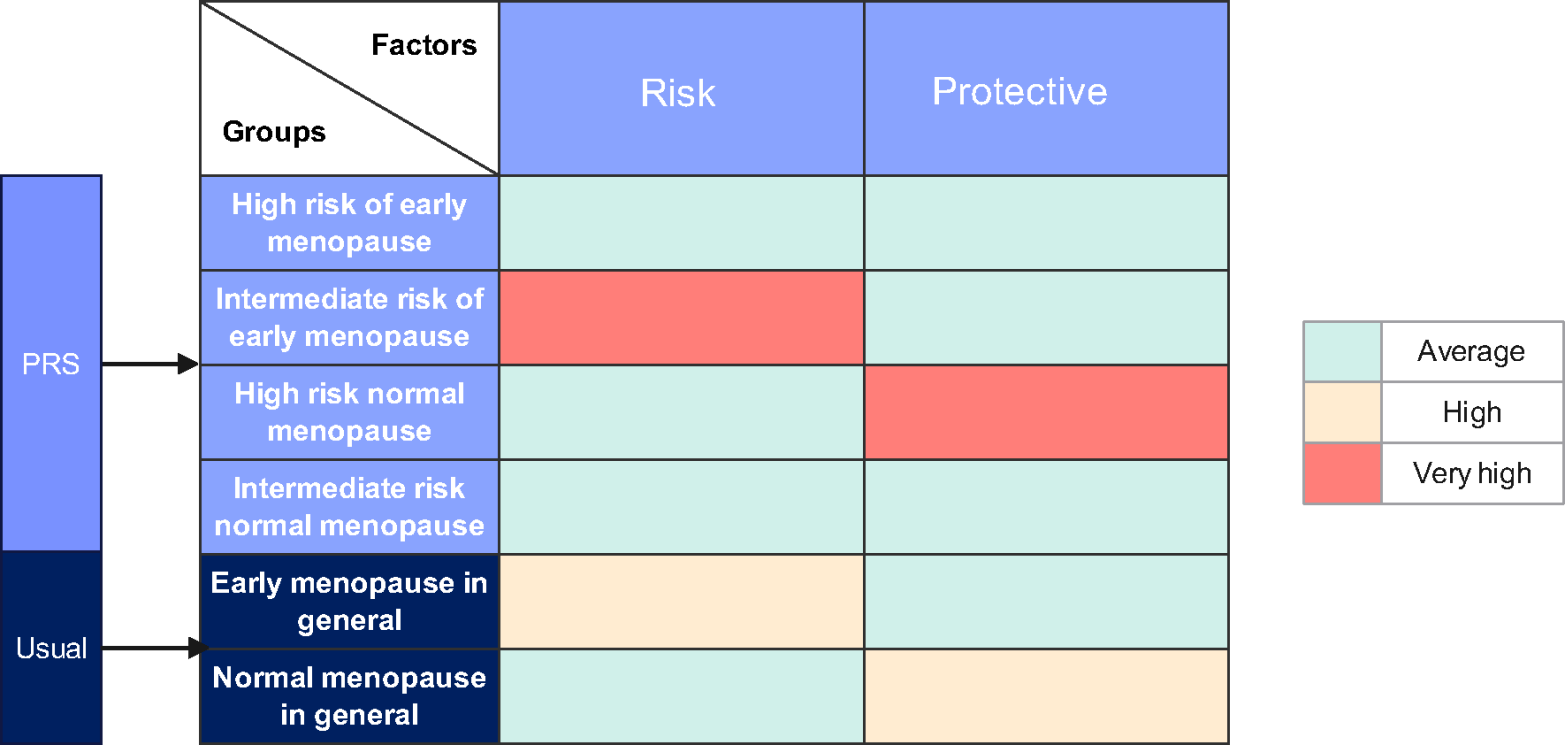
The distribution of factors affecting early menopause across various populations. The population experiencing normal menopause at Intermediate risk served as the control group, with the prevalence of each risk and protective factor in this group considered average. This establishes a baseline against which the influence of these factors in other populations can be measured. Given that the proportions of influencing factors in the population at high risk for early menopause mirror those in the control group, these similarities allow for the high-risk group to be used as a comparative control in further exploration of early menopause risk factors.

If a threshold is established within the PRS model for classification, women with a high genetic predisposition to EM could be considered high risk, while others might be categorized as intermediate risk, contingent on research protocols. Furthermore, the female population can be divided into four groups according to their menopausal status. These groups are theoretically exposed to varying degrees of risk and protective factors (Events that delay the onset) for menopause (**Figure 2**). It is also notable that women at high genetic risk for EM are typically those experiencing genetically-influenced EM without a significant number of protective factors. They exhibit a ratio of risk and protective factors comparable to that of the general population, namely women at intermediate risk for regular menopause. Conversely, women at intermediate risk for EM are frequently exposed to a greater number of risk factors than those with genetically-induced EM, and are subjected to a higher proportion of risk factors than the general population, while maintaining comparable levels of protective factors. At present, there is a paucity of research characterizing women at high and intermediate risk for EM. Given that the factors influencing women at high risk are comparable to those affecting women at intermediate risk for regular menopause, the former can be utilized as an approximate substitute for the latter group. This substitution allows for a comparative analysis with the intermediate risk EM cohort, enabling the investigation of risk factors for EM and enhancing statistical efficacy within a limited sample size.

## 3 Results

### 3.1 EM PRS model establishment and validation

**Figure 3:**
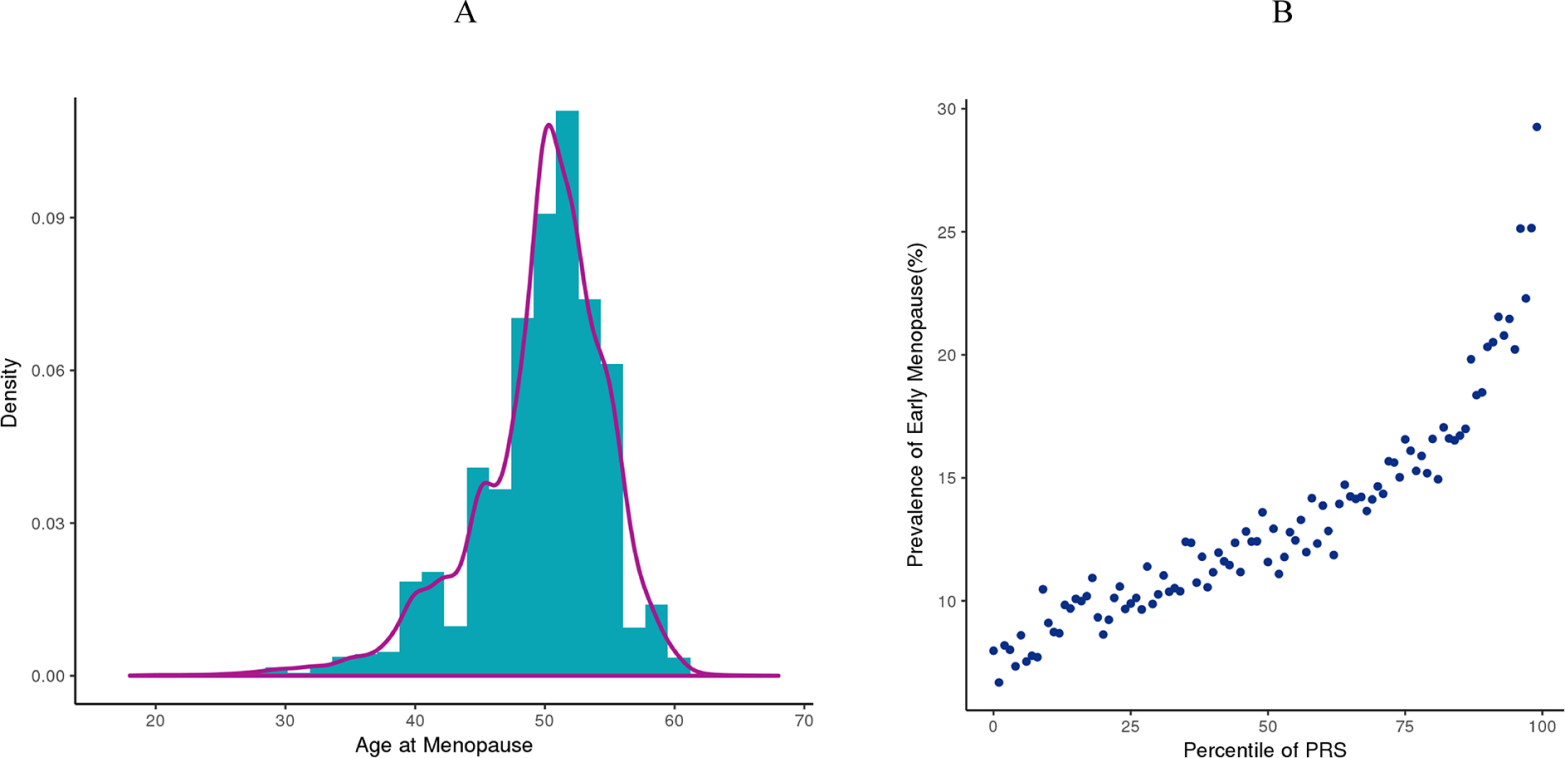
Early menopause PRS model establishment based on UK Biobank cohort. **(A)** Distribution of age at menopause from women in the UK Biobank dataset. **(B)** Prevalence of women with EM at each PRS percentile in the UK Biobank dataset.

We developed an EM PRS model including 290 SNPs derived from the previous GWAS using approximately 200,000 women of European ancestry (7). To establish EM PRS model, 154,320 women with known age of menopause derived from UK Biobank were included. The distribution of age of menopause of these women were shown in **Figure 3A**, with a peak around 50 years old. Approximately 13% (20,408) women were with EM. Using genotyping microarray data of all these women, we calculated EM PRS for each individual and found that the prevalence of women with EM were increasingly higher along with larger PRS percentile (**Figure 3B**).

**Figure 4:**
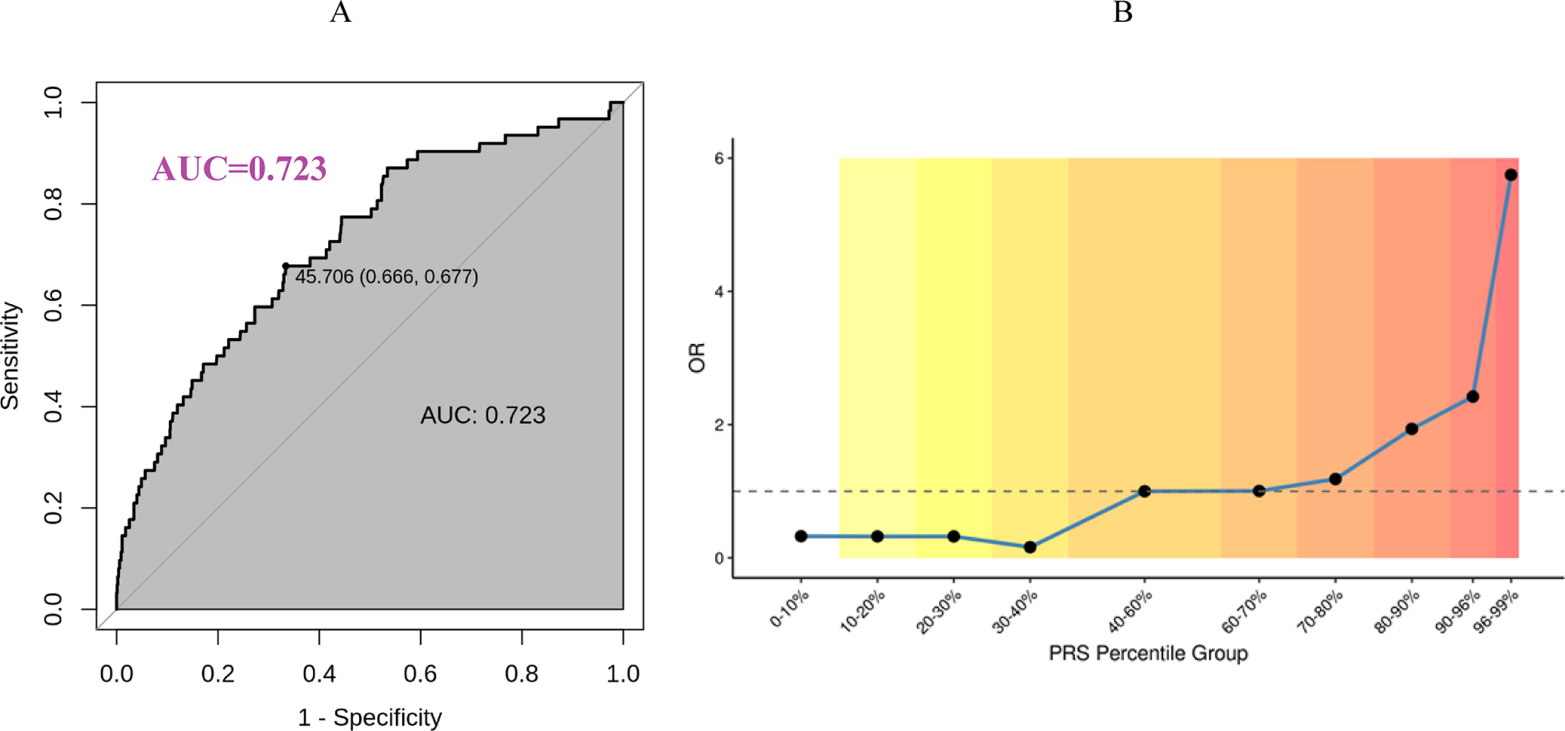
Early menopause PRS model validation based on Chinese cohort. **(A)** EM PRS model performance in Chinese cohort. **(B)** Predicted EM risk at different PRS percentile group in Chinese cohort.

For EM PRS model validation, we recruited an independent Chinese cohort with 99 women with EM and 1027 women as controls. As revealed by **Figure 4A**, the AUC of EM PRS model was 0.723 in Chinese cohort, indicating excellent performance of EM PRS model in Chinese cohort. Moreover, according to the ROC curve in **Figure 4A**, we defined the PRS value of 45.706 as the cut-off to determine whether a woman was with EM high risk. If a woman with a PRS lower than 45.706, this woman was predicted with EM high risk. Accordingly, a PRS value of 45.706 was equal to a PRS percentile of 90% based on the PRS distribution of the Chinses cohort. That is, a woman at a PRS percentile higher than 90% was considered with EM high risk. The association between the EM PRS and EM risk by percentiles are shown in **Figure 4B**. Compared to women with average risk (40-60% percentiles), the observed OR of developing EM for women in the highest 5% of the EM PRS distribution was 5.75.

### 3.2 Different risk classifications of EM PRS model in different groups of women

To date, the study has enrolled 99 eligible women with early menopause or POF, two of whom had unexplained primary amenorrhea. As shown in **Table 1**, among the 99 women recruited with EM, the proportion classified as high risk by the PRS model was 36.4% (36/99), while the intermediate-risk group constituted 63.6% (63/99). In contrast, among the 1027 patients in the PGT-M database, the high-risk group was 13.1% (135/1027), and the intermediate-risk group was 86.9% (892/1027). The high-risk proportion between the EM group and the PGT-M group differed significantly (*p*< 0.01, OR = 3.78).

**Table 1:** Differences in the proportion of high-risk PRS in the early menopausal population compared with the PGT-M group and the normal menopausal group.

	High risk	Intermediate risk	χ²*	P*	OR*
Early menopausal	36 (36.4%)	63 (63.6%)	/	/	/
PGT-M	135 (13.1%)	892 (86.9%)	37.79	<0.001	3.78(2.41-5.91)
Normal menopause (UK Biobank)	37 (10.1%)	331 (89.9%)	40.94	<0.001	5.11(3.00-8.70)

*Comparison with Early Menopause.

Similarly, in the UK Biobank database, the proportion of high-risk Chinese women with normal menopause was 10.1% (37/368), and the intermediate-risk group was 89.9% (331/368). The high-risk proportion in the normal menopause group was significantly lower than in the early menopause group, as confirmed by the chi-square test (*p*< 0.01, OR = 5.11). This suggests that women with a high-risk PRS score are approximately 5.11 times more likely to experience EM compared to those with an intermediate-risk PRS score. score.

### 3.3 Characteristics of different genetic risk groups in women with early menopause

As shown in **Table 2**, there are significant differences in certain variables between high-risk and intermediate-risk early menopausal women, indicating that the demographic and clinical characteristics of the two groups are inconsistent.

**Table 2:** Comparison of characteristics of high-risk and intermediate-risk genetic groups in women with early menopause (positive results).

Variables	Total (n = 99)	High risk (n = 36)	Intermediate risk (n = 63)	Statistic	*P*
Height(cm), Mean ± SD	162.08 ± 5.50	160.64 ± 4.57	162.90 ± 5.84	t=-1.99	0.049
Family satisfaction score, n(%)				–	0.024
Dissatisfied	4 (4.04)	0 (0.00)	4 (6.35)		
Average	12 (12.12)	6 (16.67)	6 (9.52)		
Relatively satisfied	62 (62.63)	27 (75.00)	35 (55.56)		
Very satisfied	21 (21.21)	3 (8.33)	18 (28.57)		
Premenopausal COVID-19 vaccination, n(%)				χ²=5.49	0.019
No	14 (14.14)	9 (25.00)	5 (7.94)		
Yes	85 (85.86)	27 (75.00)	58 (92.06)		
Family history of early menopause, n(%)				χ²=4.71	0.030
No	80 (80.81)	25 (69.44)	55 (87.30)		
Yes	19 (19.19)	11 (30.56)	8 (12.70)		
The husband smokes, n(%)				χ²=9.96	0.002
No	56 (60.22)	27 (81.82)	29 (48.33)		
Yes	37 (39.78)	6 (18.18)	31 (51.67)		
The husband drinks heavily, n(%)				χ²=14.67	<.001
No	57 (61.96)	29 (87.88)	28 (47.46)		
Yes	35 (38.04)	4 (12.12)	31 (52.54)		
Habit of staying up late, n(%)				χ²=4.34	0.037
No	24 (24.24)	13 (36.11)	11 (17.46)		
Yes	75 (75.76)	23 (63.89)	52 (82.54)		

t, t-test; χ², Chi-square test; -, Fisher exact; SD, standard deviation. Due to the large amount of survey information collected, only positive results are presented in the main text. For a complete analysis of the results, please refer to **Supplementary Table 1**.

Specifically, compared with women at intermediate risk of early menopause, women at high risk exhibited the following differences:

1. Height: High-risk women were shorter (160.64 cm vs. 162.90 cm, *p* = 0.049).
2. Family Satisfaction: A lower proportion of high-risk women reported being very satisfied with their families (8.33% vs. 28.57%, *p* = 0.024).
3. Premenopausal COVID-19 Vaccination: Fewer high-risk women had received the COVID-19 vaccine (75.00% vs. 92.06%, *p* = 0.019).
4. Family History: A higher proportion of high-risk women had a family history of early menopause (30.56% vs. 12.70%, *p* = 0.030).
5. Staying Up Late: A lower proportion of high-risk women reported staying up late (63.89% vs. 82.54%, *p* = 0.037).
6. The husband smokes: A lower proportion of spouses of high-risk women smoked (18.18% vs. 58.67%, *p* = 0.002).
7. The husband drinks heavily: A lower proportion of spouses of high-risk women drank alcohol heavily (12.12% vs. 52.54%, *p*< 0.001).

These findings suggest that high-risk women for early menopause have distinct demographic and clinical profiles compared to their intermediate-risk counterparts.

### 3.4 Risk factors for early menopause

As shown in **Figure 2**, the proportion of influencing factors in the high-risk premature menopausal population is similar to that of the intermediate-risk normal menopausal population. Therefore, when studying risk factors for early menopause, the high-risk premature menopausal population can be approximately treated as the intermediate-risk normal menopausal population. The differences in characteristics of women with varying genetic risks are ultimately differences in the risk factors for early menopause they face.

#### 3.4.1 Risk factors for early menopause

Variables such as being very satisfied with the family, receiving the Premenopausal COVID-19 vaccine, staying up late, and smoking and drinking heavily by the male partner have a higher proportion in the intermediate-risk group of early menopause. These variables should be considered risk factors for promoting early menopause.

#### 3.4.2 Established characteristics of high-risk early menopausal women

The family history of early menopause was more prevalent in high-risk early menopausal women. These should be regarded as established characteristics of high-risk early menopausal women.

#### 3.4.3 Height

Since height is a measurement index and not an external factor, it cannot be simply classified as a risk factor or established characteristic based on its value.

#### 3.4.4 Non-significant risk factors

There were no significant differences between the two groups in traditional risk factors for POI, such as female smoking, passive smoking, chickenpox, and mumps.

## 4 Discussion

This study recruited women with EM from multiple centers in China, utilizing data from the UK Biobank and our own PGT-M patient database. Women were categorized into different genetic susceptibility groups for EM based on the PRS model. The findings demonstrated that the PRS model is an effective tool for predicting EM. Furthermore, the study explored the differences in various characteristics between high-risk and intermediate-risk EM groups and analyzed the risk factors. The study provides valuable insights into the application of the PRS model in women with EM and offers a more accurate analysis of the risk factors associated with EM.

People have gradually come to realize that a combination of genetic predispositions and external factors can induce many diseases, including EM. Utilizing PRS to categorize women who have experienced EM can help explain to patients to what extent their condition may be attributed to genetic predispositions versus acquired factors.

Recent research on PRS models for EM has shown promising results. PRS constructed from genetic variants associated with age at menopause and menarche have demonstrated significant associations with reproductive timing and hormone levels across multiple ethnic groups (8). These scores can potentially estimate an individual’s lifetime genetic risk of EM, although their current discriminative ability in the general population remains low (9). Genome-wide association studies have identified numerous candidate genes and loci associated with EM, advancing our understanding of its genetic factors (10). However, a systematic review of risk prediction models for natural menopause onset revealed limitations in their performance and generalizability, with most studies focusing on Caucasian populations and exhibiting high risk of bias (11). Further research is needed to improve the accuracy and applicability of these models across diverse populations.

The verified data for this study were derived from Asian women, revealing that the proportion of women with a high genetic risk of PRS experiencing EM is 5.11 times that of intermediate-risk women. When the Chinese PGT-M population (using non-public data) served as the control group, the calculated OR was 3.58. This finding provides further support for the predictive validity of the PRS tool. This study not only addresses a gap in existing research but also demonstrates that PRS is a significant predictor of genetic risk for EM. Although the PRS tool and the determination of genetic risk thresholds have inherent limitations, the primary objective remains the identification of individuals with a high genetic risk of EM for effective risk stratification. This information could be valuable for making informed decisions regarding participation in screening programmes, lifestyle modifications, or fertility preservation.

After stratifying the recruited women with EM based on genetic risk, we identified distinct characteristics between those in different genetic risk groups. These differences partly stem from the inconsistency in the external risk factors they are exposed to. Theoretically, women at intermediate risk for early menopause are more likely to encounter adverse social and environmental factors (**Figure 2**). Additionally, some differences in characteristics can be attributed to the established traits of high-risk patients, such as a family history of EM.

It is understandable that the proportion of women with a family history of EM is higher in the high genetic risk group (30.56% vs. 12.70%, *p*=0.030). This is because PRS effectively identifies women with high genetic risk, and their high-risk gene loci are inherited from both parents. Specifically, women at high risk are approximately 2.4 times more likely to have a family history of the condition compared to those at intermediate risk. This finding underscores the significant role of genetic factors in the occurrence of EM and suggests that female offspring of the high-risk group identified by PRS are at an increased risk of experiencing EM. Patients should be fully informed of these potential risks and advised to plan the timing of childbearing accordingly for the women in their families.

Additionally, we observed that women with EM in the high-risk group were shorter on average than those in the intermediate-risk group (160.64 cm vs. 162.90 cm, *p*=0.049). This may be due to some of the genetic loci associated with EM also affecting growth and development. Previous studies have indicated that higher height is associated with delayed menopause, supporting our findings that genetic factors influencing height may also play a role in the timing of menopause (12).

In this study, variables such as high family satisfaction ratings, Premenopausal COVID-19 vaccination, staying up late, male smoking, and drinking heavily were found to be more prevalent in the intermediate-risk early menopause group. These factors can thus be considered potential risk factors for EM.

Previous research has demonstrated that family-related factors, particularly marital satisfaction, impact menopausal symptoms in middle-aged women (13). However, there is a lack of research on the relationship between family satisfaction and the risk of EM. Our study suggests that high family satisfaction ratings may be a risk factor for EM. This could be due to the fact that most family relationships are imperfect, and the majority of volunteers in this study were relatively satisfied with their family ratings. Excessive satisfaction might indicate that these women engage in behaviors detrimental to ovarian function, such as staying up late, smoking, and drinking. Nevertheless, it is challenging to directly explain how excessive family satisfaction affects the risk of EM.

Moreover, our study found that Premenopausal COVID-19 vaccination may be a risk factor for EM. Recent studies have examined the potential impact of COVID-19 vaccination on the menstrual cycle, with some women reporting changes in menstrual patterns post-vaccination. However, these changes are generally temporary and do not indicate EM (14, 15). It is noteworthy that COVID-19 infection itself is associated with a higher risk of menstrual disorders than vaccination alone (15). While these studies acknowledge that menstrual changes can occur after vaccination, especially among those with a confirmed history of COVID-19 infection before EM, the potential side effects of the vaccine and the virus itself may contribute to premature ovarian function decline. However, given that COVID-19 vaccination policies can introduce many confounding factors, the conclusions of this research are subject to potential biases.

Similar to previous studies, our research also emphasizes the harmful effects of staying up late or working night shifts on ovarian function. Research suggests that night shift work may accelerate the onset of EM, particularly in younger women. A study found that nurses under 45 working rotating night shifts had a higher risk of EM (16). The association between night work and reproductive health is thought to be mediated by circadian disruption, which can affect melatonin production and ovarian hormone levels (17). However, the evidence is not conclusive, and limitations exist in the measurement of night work exposure across studies (18). The impact of night work on menstrual cycles, fertility, and pregnancy has been investigated, but research specifically addressing menopause is limited (18). While night shift work may modestly accelerate reproductive senescence in predisposed women, more studies are needed to fully understand the relationship between night work and menopausal timing (16, 17).

Research suggests that smoking is significantly associated with EM (19). And a meta-analysis confirmed that smoking is a significant independent factor for EM (20). In our study, the proportion of female smokers did not differ significantly between the two groups, likely due to the lower prevalence of smoking among Chinese women. However, the results indicate that male smoking may be a risk factor for EM in women. This is likely due to the early decline in ovarian function caused by long-term passive smoking.

Research has shown that moderate alcohol consumption may be associated with delayed menopause. For example, Freeman et al. (21) found that women who consumed 10-14.9 g of alcohol per day had a lower risk of early menopause compared to non-drinkers. Similarly, Tanari et al. (22) reported that low to moderate alcohol intake (0-16 g/day) was associated with delayed menopause. The relationship between alcohol and the onset of menopause may also be influenced by the type of alcoholic beverage, with white wine, red wine, and spirits showing a stronger association than beer (21). It is important to note that heavy drinking does not appear to be associated with a reduced risk of early menopause (21). Consequently, there is no strong evidence that alcohol intake is a risk factor for EM. Our study also did not find that alcohol intake in women was a risk factor. However, our results suggest that male drinking heavily may be a risk factor for EM in women. Similar to the findings related to family satisfaction, it is difficult to directly explain how men’s drinking heavily is a risk factor for their wives’ EM. We speculate that women married to men who drink heavily may stay up late and have shorter sleep durations due to their husbands’ frequent night drinking parties, and may also experience psychological stress, which could lead to a faster decline in ovarian function.

Therefore, unlike most previous studies that focus solely on women, our study emphasizes that certain behaviors of husbands within the family may adversely affect their wives’ ovarian function. This further highlights that EM in women involves complex social issues.

In summary, predicting EM is challenging, and identifying its risk factors is complicated by various confounding factors and biases. This study innovatively used the PRS for EM to stratify women with different genetic risks. This approach not only validated the predictive efficacy of the PRS tool but also revealed characteristic differences among women with varying genetic risks of EM, further exploring the associated risk factors.

Certainly, this study has some inevitable limitations. Firstly, the EM PRS tool, established based on European and American populations, may not be entirely suitable for Asian populations. However, it remains the most appropriate tool available for our use so far. Secondly, the PRS risk threshold, set at above 90%, might not be optimal, and future studies should aim to determine a more suitable threshold. Lastly, the questionnaires used in this study were retrospective and self-administered by the patients, raising concerns about the authenticity of the responses. Failure to recruit patients to establish a rigorously defined normal menopausal control group also introduced bias into the exploration of risk factors in this study. It is essential to rigorously match the control population with those experiencing normal menopause to further validate the clinical value of the PRS tool. Robust experimental design is crucial for ensuring the reliability of the results, which necessitates further efforts from scholars to enhance experimental design and allocate more resources based on our work.

Unquestionably, our findings encourage further studies utilizing the EM PRS tool to stratify women by genetic risk, thereby enhancing efforts to protect women’s fertility. The observed differences in characteristics among women with varying genetic risks offer new insights for future research on the impact of EM gene risk loci on body structure and function. Additionally, employing more statistically robust methods in future studies will help explore risk factors for EM, even with limited sample sizes. This is crucial for the primary prevention of EM in women.

## Data Availability

The raw data supporting the conclusions of this article will be made available by the authors, without undue reservation.

## Supplementary material

Supplementary Material S1 Comparison of characteristics of high-risk and intermediate-risk genetic groups in women with early menopause.
