# Correction: Genetic risk stratification and risk factors of early menopause in women: a multi-center study utilizing polygenic risk scores

**DOI:** 10.3389/fendo.2026.1778205

**Published:** 2026-02-11

**Authors:** Wei Zhong, Qihang Wang, Dingchuan Peng, Yangyun Zou, Yulin Chen, Yingying Xia, Xin Zhang, Mingming Shu, Chunlan Song, Yiran Wang, Yiyao Fu, Sishuo Wang, Yanmin Ma, Xiaomeng Bu, Yuexiu Liang, Yuzhen Chen, Wenpei Bai, Yanrong Chen, Chengyan Deng, Wanyu Zhang, Ming Zhou, Lijuan Lv, Linyan Zhang, Sijia Lu, Wei Shang

**Affiliations:** 1 Department of Obstetrics and Gynecology, The Seventh Medical Center of People’s Liberation Army General Hospital, Beijing, China; 2 Department of Obstetrics and Gynecology, The Sixth Medical Center of People’s Liberation Army General Hospital, Beijing, China; 3 Department of Obstetrics and Gynecology, Beijing Obstetrics and Gynecology Hospital, Capital Medical University, Beijing, China; 4 Department of Clinical Research, Yikon Genomics Co., Ltd, Suzhou, Jiangsu, China; 5 Department of Obstetrics and Gynecology, Chinese People’s Liberation Army Medical School, Beijing, China; 6 Department of Obstetrics and Gynecology, Affiliated Hospital of Youjiang Medical University of Nationalities, Baise, Guangxi, China; 7 Department of Obstetrics and Gynecology, BeiJing Shijitan Hospital, Beijing, China; 8 Department of Obstetrics and Gynecology, Peking Union Medical College Hospital, Beijing, China; 9 Department of Obstetrics and Gynecology, Huanghua Development Zone Boai Hospital, Cangzhou, Hebei, China; 10 Department of Obstetrics and Gynecology, Maternal and Child Health Hospital of Zhuji, Zhuji, Zhejiang, China

**Keywords:** early menopause, premature ovarian failure, polygenic risk scores, premature ovarian insufficiency, multi-center

In the **Abstract**, the word “higher” was erroneously used instead of “shorter” in the **Conclusions** section. The corrected sentence appears below.

“The exploratory analysis revealed that women with high risk of EM exhibited a shorter height, suggesting EM related genetic loci may also influence growth and development level.”

The original version of this article has been updated.

