# Supplementary material for: Genetic risk stratification and risk factors of early menopause in women: a multi-center study utilizing polygenic risk scores
Source: Front Endocrinol (Lausanne). 2024 Dec 2;15:1518288. doi: 10.3389/fendo.2024.1518288 (PMC11646720; doi:10.3389/fendo.2024.1518288)
Supplement: Supplementary Material S1 — Comparison of characteristics of high-risk and intermediate-risk genetic groups in women with early menopause. [file Table1.docx]

### Supplement 1：Comparison of characteristics of high-risk and intermediate-risk genetic groups in women with early menopause

| Variables | Total (n = 99) | High risk (n = 36) | Medium risk (n = 63) | Statistic | *P* |
| --- | --- | --- | --- | --- | --- |
|  |  |  |  |  |  |
| Age, Mean ± SD | 36.14 ± 6.71 | 37.03 ± 6.48 | 35.63 ± 6.84 | t=0.99 | 0.323 |
| Menarche age, Mean ± SD | 13.03 ± 1.68 | 12.97 ± 1.50 | 13.06 ± 1.78 | t=-0.26 | 0.795 |
| Age of menopause, Mean ± SD | 31.60 ± 7.49 | 32.34 ± 7.69 | 31.18 ± 7.40 | t=0.73 | 0.464 |
| Number of pregnancies, Mean ± SD | 1.15 ± 1.68 | 1.17 ± 1.44 | 1.14 ± 1.81 | t=0.07 | 0.946 |
| Height(cm), Mean ± SD | 162.08 ± 5.50 | 160.64 ± 4.57 | 162.90 ± 5.84 | t=-1.99 | **0.049** |
| Weight(kg), Mean ± SD | 61.24 ± 15.63 | 62.52 ± 16.45 | 60.51 ± 15.23 | t=0.61 | 0.541 |
| Body Mass Index (BMI), Mean ± SD | 23.36 ± 6.09 | 24.24 ± 6.33 | 22.85 ± 5.93 | t=1.10 | 0.275 |
| TSH （MIU/L）, Mean ± SD | 2.28 ± 1.31 | 2.34 ± 1.04 | 2.24 ± 1.44 | t=0.33 | 0.739 |
| FSH (mIU/ml), Mean ± SD | 66.00 ± 36.39 | 60.80 ± 36.89 | 68.89 ± 36.08 | t=-1.05 | 0.294 |
| （AMH）（ng/ml）, Mean ± SD | 0.06 ± 0.14 | 0.05 ± 0.09 | 0.07 ± 0.16 | t=-0.82 | 0.412 |
| Nation, n(%) |  |  |  | χ²=1.17 | 0.280 |
| Han | 91 (91.92) | 35 (97.22) | 56 (88.89) |  |  |
| National minority | 8 (8.08) | 1 (2.78) | 7 (11.11) |  |  |
| Census register, n(%) |  |  |  | χ²=0.79 | 0.375 |
| agricultural | 33 (33.33) | 10 (27.78) | 23 (36.51) |  |  |
| non-agricultural | 66 (66.67) | 26 (72.22) | 40 (63.49) |  |  |
| Female education background, n(%) |  |  |  | χ²=2.98 | 0.394 |
| Associate's degree | 12 (12.12) | 7 (19.44) | 5 (7.94) |  |  |
| Bachelor's degree | 50 (50.51) | 17 (47.22) | 33 (52.38) |  |  |
| Below associate's degree | 20 (20.20) | 6 (16.67) | 14 (22.22) |  |  |
| Master's degree | 17 (17.17) | 6 (16.67) | 11 (17.46) |  |  |
| Education background of husband, n(%) |  |  |  | χ²=2.01 | 0.570 |
| Associate's degree | 18 (19.35) | 6 (18.75) | 12 (19.67) |  |  |
| Bachelor's degree | 44 (47.31) | 16 (50.00) | 28 (45.90) |  |  |
| Below associate's degree | 23 (24.73) | 9 (28.12) | 14 (22.95) |  |  |
| Master's degree | 8 (8.60) | 1 (3.12) | 7 (11.48) |  |  |
| Previous menstrual cycles, n(%) |  |  |  | χ²=0.99 | 0.320 |
| irregular | 30 (30.93) | 13 (37.14) | 17 (27.42) |  |  |
| regular | 67 (69.07) | 22 (62.86) | 45 (72.58) |  |  |
| Days of menstrual bleeding, n(%) |  |  |  | - | 0.113 |
| >10 Days | 3 (3.09) | 1 (2.86) | 2 (3.23) |  |  |
| ≤7 Days | 75 (77.32) | 31 (88.57) | 44 (70.97) |  |  |
| 7-10 Days | 19 (19.59) | 3 (8.57) | 16 (25.81) |  |  |
| Previous menstrual flow, n(%) |  |  |  | χ²=0.04 | 0.981 |
| heavy | 17 (17.53) | 6 (17.14) | 11 (17.74) |  |  |
| low | 13 (13.40) | 5 (14.29) | 8 (12.90) |  |  |
| normal | 67 (69.07) | 24 (68.57) | 43 (69.35) |  |  |
| Menopause, n(%) |  |  |  | χ²=0.53 | 0.465 |
| No | 32 (32.32) | 10 (27.78) | 22 (34.92) |  |  |
| Yes | 67 (67.68) | 26 (72.22) | 41 (65.08) |  |  |
| Age of first sexual intercourse, n(%) |  |  |  | χ²=0.08 | 0.962 |
| <20 | 10 (10.10) | 4 (11.11) | 6 (9.52) |  |  |
| >25 | 41 (41.41) | 15 (41.67) | 26 (41.27) |  |  |
| 20~25 | 48 (48.48) | 17 (47.22) | 31 (49.21) |  |  |
| Marital relationship, n(%) |  |  |  | - | 0.512 |
| average | 36 (37.89) | 12 (36.36) | 24 (38.71) |  |  |
| Bad | 5 (5.26) | 3 (9.09) | 2 (3.23) |  |  |
| harmony | 54 (56.84) | 18 (54.55) | 36 (58.06) |  |  |
| Sexual life, n(%) |  |  |  | χ²=2.90 | 0.235 |
| average | 51 (52.58) | 18 (52.94) | 33 (52.38) |  |  |
| Bad | 13 (13.40) | 7 (20.59) | 6 (9.52) |  |  |
| Good | 33 (34.02) | 9 (26.47) | 24 (38.10) |  |  |
| Female smoke, n(%) |  |  |  | χ²=0.36 | 0.550 |
| No | 93 (93.94) | 35 (97.22) | 58 (92.06) |  |  |
| Yes | 6 (6.06) | 1 (2.78) | 5 (7.94) |  |  |
| Heavy drinking, n(%) |  |  |  | χ²=0.58 | 0.448 |
| No | 86 (86.87) | 33 (91.67) | 53 (84.13) |  |  |
| Yes | 13 (13.13) | 3 (8.33) | 10 (15.87) |  |  |
| Coarse grains, n(%) |  |  |  | χ²=2.64 | 0.267 |
| always | 22 (22.22) | 5 (13.89) | 17 (26.98) |  |  |
| hardly | 8 (8.08) | 4 (11.11) | 4 (6.35) |  |  |
| normal | 69 (69.70) | 27 (75.00) | 42 (66.67) |  |  |
| High-protein diet, n(%) |  |  |  | χ²=0.14 | 0.704 |
| always | 71 (71.72) | 25 (69.44) | 46 (73.02) |  |  |
| normal | 28 (28.28) | 11 (30.56) | 17 (26.98) |  |  |
| Obesity, n(%) |  |  |  | χ²=0.62 | 0.431 |
| No | 81 (81.82) | 28 (77.78) | 53 (84.13) |  |  |
| Yes | 18 (18.18) | 8 (22.22) | 10 (15.87) |  |  |
| History of vaginitis, n(%) |  |  |  | χ²=0.36 | 0.549 |
| No | 65 (65.66) | 25 (69.44) | 40 (63.49) |  |  |
| Yes | 34 (34.34) | 11 (30.56) | 23 (36.51) |  |  |
| History of cervicitis, n(%) |  |  |  | χ²=0.01 | 0.918 |
| No | 83 (83.84) | 30 (83.33) | 53 (84.13) |  |  |
| Yes | 16 (16.16) | 6 (16.67) | 10 (15.87) |  |  |
| History of pelvic inflammatory disease, n(%) |  |  |  | χ²=1.46 | 0.227 |
| No | 82 (82.83) | 32 (88.89) | 50 (79.37) |  |  |
| Yes | 17 (17.17) | 4 (11.11) | 13 (20.63) |  |  |
| Uterine fibroids, n(%) |  |  |  | χ²=1.33 | 0.248 |
| No | 93 (93.94) | 32 (88.89) | 61 (96.83) |  |  |
| Yes | 6 (6.06) | 4 (11.11) | 2 (3.17) |  |  |
| Endometriosis, n(%) |  |  |  | χ²=0.32 | 0.574 |
| No | 90 (90.91) | 34 (94.44) | 56 (88.89) |  |  |
| Yes | 9 (9.09) | 2 (5.56) | 7 (11.11) |  |  |
| Infertility, n(%) |  |  |  | χ²=0.99 | 0.319 |
| No | 56 (56.57) | 18 (50.00) | 38 (60.32) |  |  |
| Yes | 43 (43.43) | 18 (50.00) | 25 (39.68) |  |  |
| Thyroid disease, n(%) |  |  |  | χ²=0.81 | 0.369 |
| No | 79 (79.80) | 27 (75.00) | 52 (82.54) |  |  |
| Yes | 20 (20.20) | 9 (25.00) | 11 (17.46) |  |  |
| Breast diseases, n(%) |  |  |  | χ²=0.01 | 0.918 |
| No | 83 (83.84) | 30 (83.33) | 53 (84.13) |  |  |
| Yes | 16 (16.16) | 6 (16.67) | 10 (15.87) |  |  |
| Diabetes, n(%) |  |  |  | χ²=0.00 | 1.000 |
| No | 94 (94.95) | 34 (94.44) | 60 (95.24) |  |  |
| Yes | 5 (5.05) | 2 (5.56) | 3 (4.76) |  |  |
| Constipation, n(%) |  |  |  | χ²=0.01 | 0.925 |
| No | 89 (89.90) | 33 (91.67) | 56 (88.89) |  |  |
| Yes | 10 (10.10) | 3 (8.33) | 7 (11.11) |  |  |
| History of chickenpox, n(%) |  |  |  | χ²=0.01 | 0.932 |
| No | 72 (72.73) | 26 (72.22) | 46 (73.02) |  |  |
| Yes | 27 (27.27) | 10 (27.78) | 17 (26.98) |  |  |
| History of mumps, n(%) |  |  |  | χ²=1.23 | 0.268 |
| No | 75 (75.76) | 25 (69.44) | 50 (79.37) |  |  |
| Yes | 24 (24.24) | 11 (30.56) | 13 (20.63) |  |  |
| History of COVID-19 infection, n(%) |  |  |  | χ²=1.47 | 0.226 |
| No | 61 (61.62) | 25 (69.44) | 36 (57.14) |  |  |
| Yes | 38 (38.38) | 11 (30.56) | 27 (42.86) |  |  |
| Skin acne, n(%) |  |  |  | χ²=2.76 | 0.097 |
| No | 88 (88.89) | 35 (97.22) | 53 (84.13) |  |  |
| Yes | 11 (11.11) | 1 (2.78) | 10 (15.87) |  |  |
| Hairy, n(%) |  |  |  | - | 0.364 |
| No | 98 (98.99) | 35 (97.22) | 63 (100.00) |  |  |
| Yes | 1 (1.01) | 1 (2.78) | 0 (0.00) |  |  |
| Lipuria, n(%) |  |  |  | χ²=0.10 | 0.754 |
| No | 91 (91.92) | 34 (94.44) | 57 (90.48) |  |  |
| Yes | 8 (8.08) | 2 (5.56) | 6 (9.52) |  |  |
| Hair loss, n(%) |  |  |  | χ²=2.44 | 0.118 |
| No | 82 (82.83) | 27 (75.00) | 55 (87.30) |  |  |
| Yes | 17 (17.17) | 9 (25.00) | 8 (12.70) |  |  |
| History of premature infants, n(%) |  |  |  | χ²=0.00 | 1.000 |
| No | 95 (95.96) | 35 (97.22) | 60 (95.24) |  |  |
| Yes | 4 (4.04) | 1 (2.78) | 3 (4.76) |  |  |
| Work stress, n(%) |  |  |  | χ²=0.01 | 0.909 |
| No | 53 (53.54) | 19 (52.78) | 34 (53.97) |  |  |
| Yes | 46 (46.46) | 17 (47.22) | 29 (46.03) |  |  |
| Work intensity, n(%) |  |  |  | - | 0.757 |
| High | 27 (27.27) | 11 (30.56) | 16 (25.40) |  |  |
| No | 2 (2.02) | 1 (2.78) | 1 (1.59) |  |  |
| normal | 70 (70.71) | 24 (66.67) | 46 (73.02) |  |  |
| Overtime, n(%) |  |  |  | - | 0.964 |
| always | 33 (33.33) | 13 (36.11) | 20 (31.75) |  |  |
| hardly | 17 (17.17) | 6 (16.67) | 11 (17.46) |  |  |
| No | 2 (2.02) | 1 (2.78) | 1 (1.59) |  |  |
| occasionally | 47 (47.47) | 16 (44.44) | 31 (49.21) |  |  |
| Keep ventilation, n(%) |  |  |  | χ²=0.30 | 0.586 |
| No | 14 (14.14) | 6 (16.67) | 8 (12.70) |  |  |
| Yes | 85 (85.86) | 30 (83.33) | 55 (87.30) |  |  |
| Family satisfaction score, n(%) |  |  |  | - | **0.024** |
| dissatisfied | 4 (4.04) | 0 (0.00) | 4 (6.35) |  |  |
| average | 12 (12.12) | 6 (16.67) | 6 (9.52) |  |  |
| relatively satisfied | 62 (62.63) | 27 (75.00) | 35 (55.56) |  |  |
| very satisfied | 21 (21.21) | 3 (8.33) | 18 (28.57) |  |  |
| HPV vaccination, n(%) |  |  |  | χ²=0.03 | 0.857 |
| No | 76 (76.77) | 28 (77.78) | 48 (76.19) |  |  |
| Yes | 23 (23.23) | 8 (22.22) | 15 (23.81) |  |  |
| Premenopausal COVID-19 vaccination, n(%) |  |  |  | χ²=5.49 | **0.019** |
| No | 14 (14.14) | 9 (25.00) | 5 (7.94) |  |  |
| Yes | 85 (85.86) | 27 (75.00) | 58 (92.06) |  |  |
| Family history of diabetes, n(%) |  |  |  | χ²=0.61 | 0.436 |
| No | 68 (68.69) | 23 (63.89) | 45 (71.43) |  |  |
| Yes | 31 (31.31) | 13 (36.11) | 18 (28.57) |  |  |
| Family history of hypertension, n(%) |  |  |  | χ²=0.01 | 0.908 |
| No | 57 (57.58) | 21 (58.33) | 36 (57.14) |  |  |
| Yes | 42 (42.42) | 15 (41.67) | 27 (42.86) |  |  |
| Family history of gynecological tumors, n(%) |  |  |  | χ²=0.31 | 0.580 |
| No | 87 (87.88) | 33 (91.67) | 54 (85.71) |  |  |
| Yes | 12 (12.12) | 3 (8.33) | 9 (14.29) |  |  |
| Family history of oligomenorrhea, n(%) |  |  |  | χ²=0.21 | 0.651 |
| No | 91 (91.92) | 32 (88.89) | 59 (93.65) |  |  |
| Yes | 8 (8.08) | 4 (11.11) | 4 (6.35) |  |  |
| Family history of infertility, n(%) |  |  |  | χ²=0.62 | 0.431 |
| No | 89 (89.90) | 34 (94.44) | 55 (87.30) |  |  |
| Yes | 10 (10.10) | 2 (5.56) | 8 (12.70) |  |  |
| Family history of recurrent abortion, n(%) |  |  |  | χ²=2.76 | 0.097 |
| No | 88 (88.89) | 29 (80.56) | 59 (93.65) |  |  |
| Yes | 11 (11.11) | 7 (19.44) | 4 (6.35) |  |  |
| Family history of early menopause, n(%) |  |  |  | χ²=4.71 | **0.030** |
| No | 80 (80.81) | 25 (69.44) | 55 (87.30) |  |  |
| Yes | 19 (19.19) | 11 (30.56) | 8 (12.70) |  |  |
| Family History of Hair Loss, n(%) |  |  |  | χ²=0.21 | 0.650 |
| No | 82 (82.83) | 29 (80.56) | 53 (84.13) |  |  |
| Yes | 17 (17.17) | 7 (19.44) | 10 (15.87) |  |  |
| The husband smokes, n(%) |  |  |  | χ²=9.96 | **0.002** |
| No | 56 (60.22) | 27 (81.82) | 29 (48.33) |  |  |
| Yes | 37 (39.78) | 6 (18.18) | 31 (51.67) |  |  |
| The husband drinks heavily, n(%) |  |  |  | χ²=14.67 | **<.001** |
| No | 57 (61.96) | 29 (87.88) | 28 (47.46) |  |  |
| Yes | 35 (38.04) | 4 (12.12) | 31 (52.54) |  |  |
| Habit of staying up late, n(%) |  |  |  | χ²=4.34 | **0.037** |
| No | 24 (24.24) | 13 (36.11) | 11 (17.46) |  |  |
| Yes | 75 (75.76) | 23 (63.89) | 52 (82.54) |  |  |
| Number of births, n(%) |  |  |  | - | 0.977 |
| 0 | 65 (65.66) | 24 (66.67) | 41 (65.08) |  |  |
| 1 | 19 (19.19) | 7 (19.44) | 12 (19.05) |  |  |
| 2 | 13 (13.13) | 4 (11.11) | 9 (14.29) |  |  |
| 3 | 2 (2.02) | 1 (2.78) | 1 (1.59) |  |  |
| t: t-test, χ²: Chi-square test, -: Fisher exact | | | | | |
| SD: standard deviation | | | | | |
